# Remarks on the Uncertain Effects and the Abuse of Iodine and Its Salts

**Published:** 1842-01-29

**Authors:** Reynell Coates


### MEDICAL EXAMINER.

NEW SERIES.

No. 5.] PHILADELPHIA, JAN. 29, 1842. [Vol. I.

### ORIGINAL COMMUNICATIONS.

*Remarks on the uncertain effects and the abuse of Iodine and its salts.* By Reynell Coates, M. D.—Under the Foreign head, in the third number of this journal, p. 48, is found an extract from the report of a discussion in the London University College Medical Society, during which evidence was given of seriously threatening effects resulting both from large and small doses of the iodide of potassium. These effects appear to render doubtful, at least, the propriety of resorting, in the first instance, and in private practice, to the full doses recently employed in the Pennsylvania Hospital, after the example of M. Ricord, (See Clinical Reports, pages 10 and 23, of this volume.) The editorial comment appended to the extract calls for further information on this subject, and the following observations,—being the substance of a note from our colleague, Dr. Biddle, may be added to the evidence of the occasional danger resulting from quantities not very unusually prescribed here and elsewhere.

Influenced in part by the Clinical Reports already referred to, Dr. Biddle very recently exhibited five grains of this iodide, four times a day, in the case of a female patient, thirty-five years of age. After thirty-five grains had been thus taken, Dr. B. was suddenly called, and found the patient "suffering from intense cephalalgia, considerable nervous disturbance, nausea, and remarkable tumefaction of the whole face." These symptoms, of a poisonous action, induced him to relinquish the use of the medicine immediately, although it had been employed in quantities much smaller than those ultimately used in the cases narrated in the Clinical Reports, and those now taken, as I am informed, by many patients in the Pennsylvania Hospital, without the production of such symptoms.

The following note, by Jas. C. L. Carson, M. D., (quoted from the N. Y. Med. Gaz., p. 333,) shows the similar influence of a much smaller dose:

"I ordered a gentleman three grains of iodide of potassium in a draught of peppermint water, three times a day. When he had taken the medicine three times, he felt poorly; and in the course of an hour after the fourth dose, he was attacked with a violent shivering fit, followed by intense headach, heat of skin, thirst, quick and very full pulse, and vomiting and purging at the same time. These symptoms were succeeded by great prostration of strength. Notwithstanding the administration of opiates and demulcents, the purging lasted for several days.

"The effects of the medicine were so violent that I have little doubt, if he had taken another dose, his life would have been forfeited. This is the only instance which I have seen of the iodide of potassium producing unpleasant effects in doses under ten grains."

It will be remembered that, in the discussion in the College Medical Society, Mr. Erichsen found the article sometimes dangerous in five grain doses, frequently repeated;—that Mr. Laskester *always* observed severe effects from *large doses!*—that Mr. Bucknill was convinced by experience that the ill consequences did not depend on the quantity given, having often witnessed them when small doses were employed;—and that Mr. Hardwicke felt assured that the varieties of effect produced did not depend upon any peculiar idiosyncracy, but upon something accidental in the condition of the patient at the moment of exhibition.

From all this evidence, it appears very clear that iodide of potassium is not injurious, *per se,* even in large doses, when properly exhibited. The degree of dilution is one of the elements to be considered in calculating its effects; for all the preparations of iodine, like iodine itself, and its kindred agents, chlorine and bromine, are exceedingly irritating when concentrated: but the remedial influence of iodine is not materially diminished by extensive dilution, while its irritating qualities are very essentially lessened by this means. Is it not equally plain that the occasional injury resulting alike from small and large doses is due to some change in the character of the remedial agent after its admission into the stomach? None of the adulterations usually resulting from the different modes in which the iodide of potassium is prepared, are of such a nature as to explain the evil influences observed, especially when the quantity administered is small. But the muriatic, the acetic, and the lactic acids—acids which are present in the stomach in various amounts—are all capable of decomposing this salt; and the immediate result of the decomposition is the elimination of the hydriodic acid. It is not probable that hydriodic acid, in moderate quantity and much diluted, can exert any dangerous effects; but whenever oxygen is present, the iodine is readily separated.

The symptoms described as attendant upon the accidental occurrence of undue action from the use of iodide of potassium are similar to those produced by free iodine; and it appears, therefore, more than probable that the presence of an unusual amount of acid in the stomach at the time of exhibition, may explain the variety of results above described, which renders somewhat uncertain and treacherous a remedy of the highest value. If this view be correct, the danger and difficulty would be removed in great degree by the previous neutralization of the acids of the stomach when considerable doses of the iodide are about to be employed. The subject is worthy the attention of some one who combines sufficient chemical knowledge with enlarged opportunities of clinical observation.

During the debate already quoted, (see London Lancet, Oct. 16, 1841, p. 96,) Mr. Hardwicke is represented as expressing doubts whether iodine has any action on the absorbents; and Mr. Erichsen referred to a case of orchitis seen by him in Paris, in which wasting of the testicle was produced by its use. The position of the former gentleman, if correctly reported, is startling; and, while we hold it utterly untenable, it is not without surprise that we see a single case of diminution of the testicle from the action of iodine or its preparations, seemingly quoted as remarkable. Can it be that this effect is so rarely witnessed in England, when, almost from the first introduction of the agent, it has been deemed necessary to caution the practitioner against the danger of such results when the plan of treatment is pushed to an immoderate extent?

We doubt whether the danger of the permanent destruction of an organ wasted by the use of such remedies, is so great as is generally supposed, even in extreme cases. A morbid deposit may be finally removed by such means ; but, when the natural structure of a gland is absorbed,—the cellular nidus still remaining,—there is at least a hope of its re-construction. A few years ago we employed preparations of iodine, both internally and externally, to an unusual extent, and with great perseverance, in the case of a young lady affected with a large recent goitre, rapidly increasing. The internal doses, at first small, were gradually increased, and, after a few weeks, the tumour began to yield very obviously; but the mammæ were diminished, seemingly, as fast as the goitre. Fearful of the destruction of the glands, the quantity exhibited internally was lessened considerably, while the local application was continued. Both the tumour and the mammæ then continued nearly stationary for some time, until the internal doses were again increased. The treatment was continued until the goitre was reduced very much, but the mammæ threatened to disappear entirely. The patient was in the first blush of womanhood, and had been rather remarkably developed. Fearing not only for the mammæ, but the ovaries, we felt compelled to state to the father the dangers that might ensue were the remedies continued, but expressed, at the same time, our conviction that the disease would ultimately yield, if he was willing to subject his daughter to the probable physiological results of the course of treatment. After some days of doubt and consultation, it was resolved that the removal of the deformity should be attempted at all hazards; and the remedies were pushed as far and as steadily as the condition of the stomach and skin and the frequent constitutional irritation would permit with any safety—farther, indeed, than strict prudence would warrant. At length the breasts became flatter than those of a child ; no glandular structure could be felt in them. The tumour continued to diminish, but less rapidly. At last it disappeared entirely. The patient was much emaciated, and we feared that her health might be permanently injured from the irritation and febrile and nervous symptoms constantly recurring during the entire spring and summer. On the cessation of the treatment, she rallied very soon, except that she continued subject to occasional nausea and vertigo. When last seen, about a year after the commencement of the treatment, her health was completely re-established, her mammæ were again fully developed, and she had completely succeeded in escaping a deformity apparently more dreaded than death, both by herself and her parents.

Residing, at that time, in a region in which goitre is not uncommon, we employed a similar though less violent course of treatment in several cases; and, though only completely successful in one other instance, the action of the remedies on the tumour and the mammæ was always more or less conspicuous.

The recent recommendation of iodine in affections of the mucous surfaces, has led to a most dangerous popular custom of employing it by the way of inhalation in affections of the throat without the advice of a physician. The article is purchased in large quantities of our apothecaries, and employed, without the slightest discretion, by persons utterly ignorant of its dangerous character; and, unless the profession should publicly discountenance this custom, there is reason to fear that we may see re-enacted follies similar to those which, on the introduction of the inhalation of etherial vapour, (twelve or fourteen years ago,) consigned a number of persons, some to the grave, some to the madhouse, and some to permanent idiocy.

